# Integrative Transcriptomic and Network Analyses Reveal Common Molecular Signatures and Turmeric‐Derived Common Therapeutic Candidates for Neurological Diseases

**DOI:** 10.1155/bmri/7855459

**Published:** 2026-11-04

**Authors:** Sumi Rani Bayragi, Riday Hossain, Md. Zohurul Islam, Sharmin Akter Sumi, Fee Faysal Ahmed

**Affiliations:** ^1^ Department of Mathematics Faculty of Science Jashore University of Science and Technology Jashore Bangladesh just.edu.bd; ^2^ High Performance Computing (HPC) Laboratory Department of Mathematics Faculty of Science Jashore University of Science and Technology Jashore Bangladesh just.edu.bd; ^3^ Department of Nursing and Health Science Faculty of Health Science Jashore University of Science and Technology Jashore Bangladesh just.edu.bd

**Keywords:** *Curcuma longa*, hub genes, integrative bioinformatics, molecular dynamics simulation, network pharmacology, neurological diseases

## Abstract

**Background:**

Neurological diseases (NDs), including Alzheimer′s disease (AD), Parkinson′s disease (PD), stroke, and brain tumors, impose a substantial global health burden. Although these disorders have distinct clinical manifestations and etiologies, accumulating evidence suggests that they share certain molecular mechanisms. However, their common molecular signatures and the therapeutic potential of turmeric‐derived bioactive compounds have not been systematically investigated.

**Objectives:**

This study is aimed at identifying shared molecular mechanisms and key hub genes across AD, PD, stroke, and glioblastoma (GBM) using an integrative bioinformatics framework and at investigating turmeric‐derived compounds as potential multitarget common therapeutic candidates.

**Materials and Methods:**

Gene expression datasets (GSE22491, GSE5281, GSE122709, and GSE4290) were retrieved from the Gene Expression Omnibus (GEO) database. Common host differentially expressed genes (cHDEGs) were identified through integrated bioinformatics analysis. Protein–protein interaction (PPI) networks were subsequently constructed to identify common hub genes (cHubGs), followed by functional enrichment and transcription factor (TF) and miRNA‐mediated regulatory network analyses. The interactions between the identified cHubGs and turmeric‐derived compounds were further investigated using molecular docking and 500‐ns molecular dynamics simulations (MDS).

**Results:**

A total of 81 cHDEGs and four cHubGs (CCNB1, CDK1, CYCS, and STAT3) were identified. Functional enrichment and regulatory network analysis revealed shared biological processes and signaling pathways, as well as key TFs and miRNAs potentially associated with ND pathogenesis. Among the 77 turmeric‐derived compounds that passed the toxicity screening, demethoxycurcumin, cyclocurcumin, and (1E,4E)‐1‐(4‐hydroxy‐3‐methoxyphenyl)‐5‐(4‐hydroxyphenyl)‐1,4‐pentadien‐3‐one exhibited the strongest predicted binding affinities. The selected compound–target complexes also showed stable behavior during the MDS.

**Conclusion:**

This study provides insights into the shared molecular features across AD, PD, stroke, and GBM and prioritizes turmeric‐derived compounds as potential multitarget therapeutic candidates. These computational findings require further validation through independent datasets and experimental studies.

## 1. Introduction

All pathological conditions that impact the nervous system are considered NDs. Biochemical, physiological, or electrical disorders in the brain, spinal cord, or peripheral nerves may result in a variety of symptoms [1]. Globally, NDs impose a substantial burden on health systems and public health. Neurologic disease was the second greatest cause of death and the primary source of disability‐adjusted life years (DALYs) worldwide in 2021 [2, 3]. The number of people who have neurologic diseases (such as AD, PD, Stroke, and brain tumors) or have died from them has increased within the last few decades [4]. A progressive NDs, AD is defined by memory loss and a number of cognitive deficits [5]. According to the 2021 International AD Report, there are around 55 million AD patients globally, and by 2050, that number is expected to grow to 152 million [6]. In the United States, AD was recorded as the underlying cause of 119,399 deaths in 2021. It was also reported as the sixth‐leading cause of death among Americans aged 65 years and older [7]. Glioblastoma (GBM), also referred to as GBM multiforme, is an aggressive primary brain tumor characterized by rapid growth, diffuse infiltration, and poor prognosis [8–11]. According to the World Health Organization (WHO), GBM is classified among high‐grade gliomas based on specific histopathological characteristics, including microvascular proliferation or tumor necrosis [12, 13]. GBM is uncommon in children and predominantly affects older adults, with a reported median age of 64 years and an incidence rate of 3.19 per 100,000 individuals in the United States [14]. Despite advances in treatment, GBM remains associated with poor survival. According to the cited source, slightly more than 5% of patients survive for 5 years, whereas approximately 0.71% survive for 10 years [15]. A common neurodegenerative disease is PD that results in significant impairment and a growing global public health burden associated with cognitive, motor, and nonmotor characteristics [16]. The frequency of PD is the second most common NDs worldwide and is gradually increasing as the population ages [17]. Around 8.51 million people worldwide suffered from PD in 2019, and as the world ages, that number is expected to increase to almost 12 million by 2040 [18, 19]. In 2016, 6.1 million individuals worldwide had PD, compared with 2.5 million in 1990. That year, PD was responsible for 3.2 million DALYs and 211,296 deaths [20]. As an atherosclerotic cardiovascular disease, the second most frequent cause of mortality is stroke and causes a high percentage of lifelong impairment, placing a significant cost on families and society [21]. The WHO calculates that 15 million individuals worldwide suffer from stroke. Of them, over 5.5 million people die from their disabilities, and approximately 5 million patients have permanent disabilities [22]. Stroke occurs at a rate of approximately 2–25 cases per 1000 people each year. The incidence of first‐ever strokes is expected to increase by approximately 30% in 2023 compared with 1983. Moreover, the risk of stroke recurrence within 5 years is estimated to range between 15% and 40% [23].

In spite of their distinct etiologies and pathological characteristics, recent research has increasingly suggested that AD, PD, GBM, and stroke may share several convergent molecular and cellular mechanisms [24–29]. AD and PD are primarily progressive neurodegenerative disorders characterized by neuronal dysfunction and loss, whereas stroke is predominantly associated with acute cerebrovascular injury, and GBM represents an aggressive primary brain malignancy characterized by uncontrolled proliferation and invasion. Nevertheless, evidence of shared pathological features has been reported among these disorders. For example, AD and PD share several features, including abnormal protein aggregation, monoaminergic dysfunction, progressive neuronal loss, and alterations in interconnected neural circuits [24, 25]. Similarly, disruption of neurovascular coupling, dysregulation of cerebral blood flow, and reactive oxygen species–mediated vascular dysfunction have been implicated in both AD and ischemic stroke [26]. In addition, transcriptomic analyses have identified shared gene deregulation between AD and GBM, particularly involving immune‐related processes and a potential chronic inflammatory state [27]. These observations suggest that, although AD, PD, stroke, and GBM are not biologically equivalent, they may converge on partially overlapping molecular and cellular processes. Therefore, comparative investigation of these disorders may help distinguish conserved molecular signatures from disease‐specific alterations and provide a broader understanding of the biological mechanisms underlying diverse neurological conditions. Such approaches have the potential to reduce polypharmacy, minimize drug–drug interactions and treatment‐related toxicity, improve patient adherence, and facilitate mechanism‐based drug repurposing, thereby accelerating therapeutic development while reducing the cost and time associated with conventional drug discovery. Natural products have emerged as promising sources of multitarget therapeutic agents because of their structural diversity and pleiotropic biological activities. Among them, *Curcuma longa* (turmeric), a medicinal plant belonging to the Zingiberaceae family, has been extensively used in traditional medicine and exhibits a wide range of pharmacological properties, including antioxidant, anti‐inflammatory, and neuroprotective activities [30–35]. Although substantial progress has been made in elucidating the molecular mechanisms of AD, PD, GBM, and stroke individually [36–43], important knowledge gaps remain. In particular, it is unclear whether common transcriptomic signatures can be systematically identified across these distinct neurological conditions and linked to potential therapeutic candidates. Previous studies have predominantly focused on individual disorders or a limited number of disease combinations, leaving the broader convergence of molecular signatures across multiple neurological conditions insufficiently explored. To address these gaps, the present study employed an integrative transcriptomic and network‐based framework to investigate AD, PD, ischemic stroke, and GBM. We aim to identify shared differentially expressed genes (DEGs) and common hub genes (cHubGs), characterize their functional and regulatory networks, and explore their potential interactions with turmeric‐derived compounds. The compounds were further evaluated using drug‐likeness and toxicity assessments, molecular docking, and molecular dynamics simulations. By integrating shared disease‐associated molecular signatures with compound‐based analyses, this study provides a systems‐level framework for prioritizing potential multitarget therapeutic candidates for further investigation.

## 2. Materials and Methods

### 2.1. Data Sources and Identification of DEGs

The human gene expression profile was taken under consideration for the purpose to investigate biological or molecular information associated to NDs. For PD, AD, stroke, and brain tumor, the gene expression profile datasets GSE22491 [44], GSE5281 [45], GSE122709 [46], and GSE4290 [47], respectively, were extracted from the GEO database (http://www.ncbi.nlm. http://nih.gov/geo/) [48]. Detailed dataset information is displayed in Table 1. The independently datasets were investigated DEGs between control samples and neurodegenerative diseases samples. The previously mentioned steps datasets were examined using the GEO2R [49] online tool, which log2‐transformed the expression matrix using the “limma” R tools to find DEGs with |log_2_FCi| > 0.5 and an adj.*p* value < 0.05. Genes with adj.*p* value < 0.05 and log_2_FCi ≥ 0.5  were considered as upregulated, and genes with adj.*p* value < 0.05 and log_2_FCi ≤ −0.5  were considered as downregulated genes.

**Table 1:** Gene′s expression profiles analyzed in this study.

GEO dataset	Platform	Diseases name	Case	Control
GSE22491	GPL6480	Parkinson	10	8
GSE5281	GPL570	Alzheimer	87	74
GSE122709	GPL20795	Ischemic stroke	10	5
GSE4290	GPL570	Brain tumor	157	23

### 2.2. Identification of Turmeric‐Derived Compounds and Their Potential Targets

We collected compounds from turmeric using IMPPAT database [50], and through these compounds, we identified potential targets. The potential targets were identified using SuperPred target prediction [51], Swiss target prediction [52], and SEA (similarity ensemble approach) [53] tools.

### 2.3. Identification of cHDEGs

To identify genes shared across the four diseases, the DEG sets obtained independently for PD, AD, stroke, and brain tumors were compared. The predicted targets of turmeric‐derived compounds were analyzed separately and subsequently used to investigate potential compound–target relationships. The identified DEGs for each disease and the genes targeted by the compounds were found to be common. These shared genes are designated as cHDEGs.

### 2.4. Network Based Molecular Interaction Analysis

To identify regulatory components and HubGs from the cHDEGs, we explored network‐based molecular interaction analysis, which included cHDEGs with TFs, miRNA, and PPI.

### 2.5. PPI and cHubGs Identification

The cHDEGs were submitted to the STRING database (https://string-db.org/) [54] to construct a protein–protein interaction (PPI) network. The resulting network was analyzed using Cytoscape and its cytoHubba plugin to identify candidate hub genes. Eight cytoHubba algorithms—betweenness, closeness, radiality, stress, degree, edge percolated component (EPC), maximum clique centrality (MCC), and maximum neighborhood component (MNC)—were applied to identify the top 10 genes according to each ranking method. The genes shared among all eight algorithms were visualized using an UpSet plot and designated as cHubGs. Multiple CytoHubba algorithms were applied to improve the robustness of hub‐gene prioritization and to reduce dependence on a single network‐ranking method.

### 2.6. Interaction Analysis of cHDEGs With Their Regulatory Factors

Regulatory biomarkers, including TFs and miRNAs, control genes at the transcriptional and posttranscriptional levels in a variety of biological processes (BP) and cellular processes [55, 56]. The TFs‐cHDEGs and miRNAs‐cHDEGs were constructed by using the databases TRRUST [57] and miRTarBase [58], respectively, through the ShinyGO [59] web‐based tool. Cytoscape [60] software was used to visualize the regulatory interaction network.

### 2.7. Functional and Pathway Enrichment Analysis of cHDEGs

For biological function and pathway enrichment analysis of cHDEGs utilizing the Gene Ontology (GO) [61] including BP, cellular component (CC), and molecular function (MF) terms was performed through Kyoto Encyclopedia of Genes and Genomes (KEGG) and the STRINGdb databases [62]. A Fisher′s exact test with a cutoff FDR value < 0.05 was used to assess the functional enrichment study.

### 2.8. Drug‐Likeness and ADMET Properties

The drug‐likeness properties of the selected compounds were evaluated using the SwissADME web server [63]. The assessment was primarily based on Lipinski′s rule of five (Ro5), including molecular weight (≤ 500 Da), lipophilicity (Log*p* ≤ 5), hydrogen bond donors (≤ 5), and hydrogen bond acceptors (≤ 10). The drug agents were taken from the PubChem database and provided in SMILES format [64]. Additional physicochemical related property, including topological polar surface area (TPSA), was also assessed. To further characterize the pharmacokinetic and metabolic properties of the selected compounds, ADMET predictions were additionally performed using the pkCSM web server [65]. The analysis included cytochrome P450 (CYP)‐related metabolic predictions, including CYP2D6 and CYP3A4 substrate status and inhibition profiles for CYP1A2, CYP2C19, CYP2C9, CYP2D6, and CYP3A4. Excretion‐related properties, particularly predicted total clearance status, were also evaluated. These computational predictions were used to provide preliminary insights into the drug‐likeness, pharmacokinetic, and metabolic profiles of the selected compounds and to prioritize candidates for subsequent toxicity assessment.

### 2.9. Prediction of Toxicity of Compounds

If an ingredient fails toxicity criteria, medication repurposing may not be successful even with strong docking results. When evaluating treatment candidates with good pharmacokinetics and safety profiles, toxicity analysis is vital to ensure that only feasible possibilities proceed ahead, particularly when assessing pharmacological compounds for potential repurposing. For these evaluations, toxicity analyses are essential and frequently take the role of conventional in vitro and in vivo research [66]. The ProTox 3.0 web server, which assesses pharmacokinetics and possible harm to the human body, was utilized to estimate the toxicity features of the drug compounds under examination [67]. Compounds were considered favorable when they were predicted to be inactive for neurotoxicity, hepatotoxicity, carcinogenicity, and cytotoxicity, while being predicted to be active for blood–brain barrier (BBB) penetration. In addition, compounds with a toxicity class > 3 and a predicted LD50 ≥ 500 mg/kg were considered to have comparatively acceptable toxicity profiles and were selected for subsequent molecular docking analysis.

### 2.10. Molecular Docking Simulation of Proteins With Aspirant Drugs

Following drug‐likeness and toxicity assessment, the prioritized turmeric‐derived compounds were subjected to molecular docking against the identified cHubGs. The three‐dimensional structures of the target proteins CDK1, STAT3, CCNB1, and CYCS were retrieved from the Protein Data Bank (PDB) using PDB IDs 6GU2, 6QHD, 4YC3, and 5EXQ, respectively [68]. Protein structures were prepared by removing water molecules, heteroatoms, and cocrystallized ligands, followed by the addition of hydrogen atoms and appropriate charges. The three‐dimensional structures of the turmeric‐derived compounds were obtained from the PubChem database in SDF format [64] and subjected to energy minimization before docking. Molecular docking was performed using PyRx Version 0.8 with AutoDock Vina [69]. The docking exhaustiveness was set to 8, and nine binding poses were generated for each docking run. The docking grid parameters were defined separately for each target protein. The grid centers and dimensions were as follows:

• CDK1: grid center X = 316.212, Y = 216.004, and Z = 192.332; dimensions X = 64.9864, Y = 46.7674, and Z = 42.7545 Å.
• CCNB1: grid center X = 27.8768, Y = −5.3921, and Z = 181.4301; dimensions X = 63.1917, Y = 71.8062, and Z = 41.8986 Å.
• CYCS: grid center X = −1.2401, Y = −3.2006, and Z = −14.3158; dimensions X = 28.5608, Y = 35.5123, and Z = 34.6661 Å.
• STAT3: grid center X = −12.481, Y = −44.890, and Z = 9.2237; dimensions X = 95.4652, Y = 62.0201, and Z = 115.9376 Å.

To assess the reproducibility of the docking results, each docking calculation was independently repeated three times under the same docking conditions, and the mean binding affinity was calculated from the three runs. Compounds with a mean binding affinity of ≤ −7.0 kcal/mol were considered for further analysis. The predicted protein–ligand interactions were visualized using Discovery Studio Visualizer [70]. The best performing compound–protein complexes were subsequently selected for molecular dynamics simulations.

### 2.11. MDS Analysis

MDS were carried out to validate the molecular docking results by evaluating the stability of the top four drug–receptor complexes over a 500‐ns trajectory. The simulations, performed with the CHARMM36 force field in Gromacs 2023 [71, 72], involved solving each complex in a TIP3P water box and neutralizing the system with ions. We employed several structural and dynamic metrics, including radius of gyration (RG), dynamic cross‐correlation matrix (DCCM), principal component analysis (PCA), root mean square deviation (RMSD), root mean square fluctuation (RMSF), solvent‐accessible surface area (SASA), secondary structure analysis (SSA), and free energy landscape analysis (FEL), to characterize the stability and conformational behavior of the complexes.

MD stability assessments were primarily based on trajectory‐derived structural and dynamic metrics, and replicate simulations were not performed; therefore, the observed differences should be interpreted cautiously.

### 2.12. Study Workflow

An overview of the entire workflow, including data collection, DEG identification, network‐based molecular interaction analysis, functional and pathway enrichment analysis, and potential drug compounds identification, is illustrated in Figure 1.

**Figure 1:**
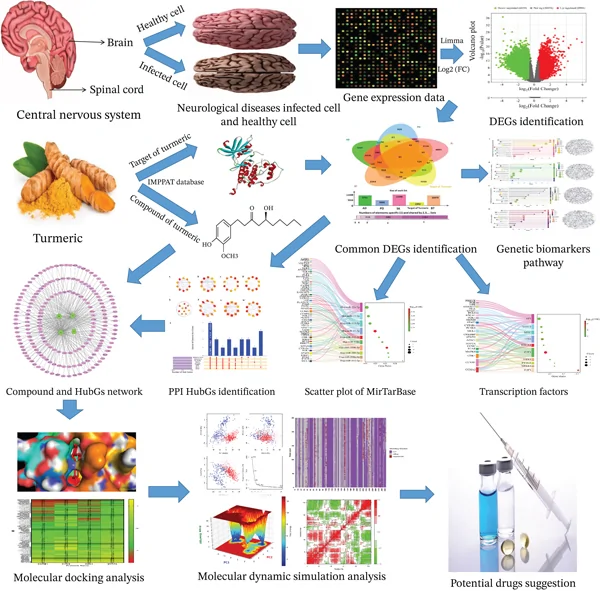
Overview of the entire study workflow.

## 3. Results

### 3.1. Identifications of DEGs in ND

In this study, the gene expression dataset of four ND datasets, that is, GSE22491 (PD), GSE5281 (AD), GSE122709 (Stroke), and GSE4290 (brain tumor), respectively, were used for the identification of DEGs, and a total of 9751, 5342, 11,048, and 10,373 DEGs were found. Figure 2 shows the upregulated, downregulated, and nonsignificant DEGs identified from the four gene expression profiles: GSE22491 (Figure 2A), GSE5281 (Figure 2B), GSE122709 (Figure 2C), and GSE4290 (Figure 2D). We find out 81 cHDEGs among the identified DEGs for NDs and target of phytochemicals, and the common DEGs among these datasets are shown using a Venn diagram (Figure 3). The Venn diagram showed 22,143 distinct DEGs, of which 0.366% were common.

**Figure 2:**
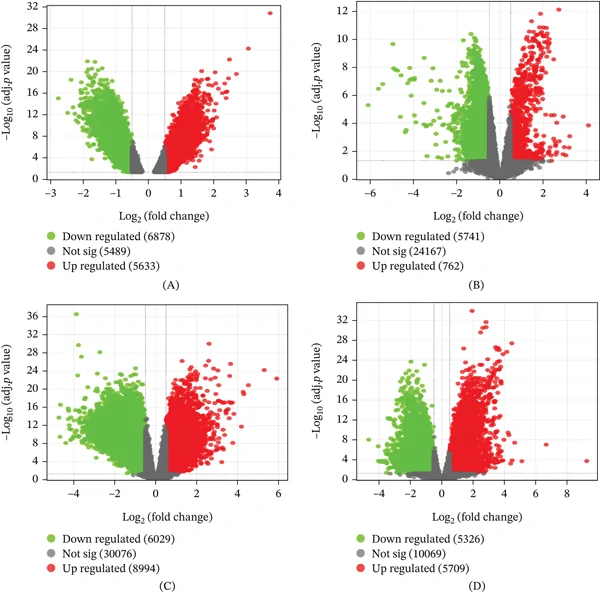
Identification of DEGs in (A) GSE22491, (B) GSE5281, (C) GSE122709, and (D) GSE4290. Red indicates upregulated genes, green indicates downregulated genes, and gray indicates nonsignificant genes.

**Figure 3:**
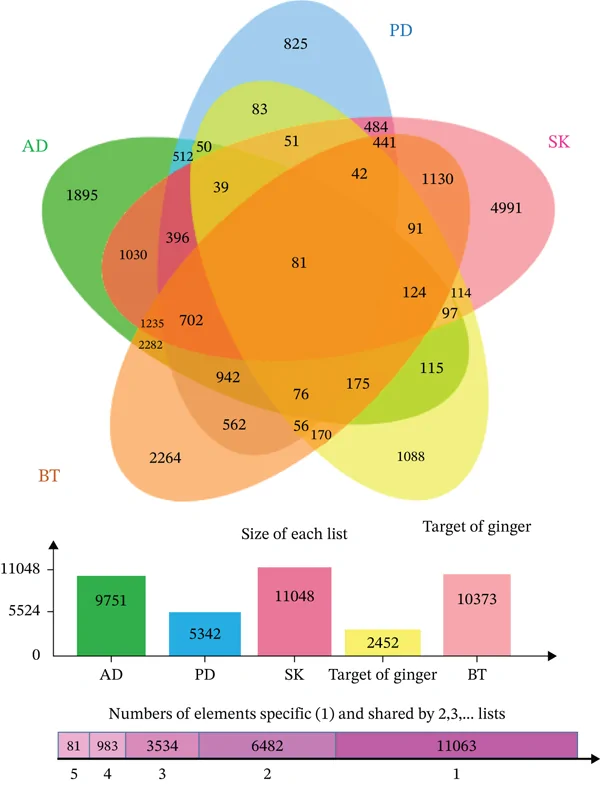
Venn diagram showing the overlap among the identified DEGs and the common genes.

### 3.2. PPI Analysis of cHDEGs

HubGs were identified by constructing a PPI network using the cHDEGs. The cHDEGs were submitted to the STRING database to retrieve their protein interaction information and construct the network, as shown in Figure 4. The top 10 HubGs were identified independently using each of the eight cytoHubba algorithms: closeness (Figure 5A), radiality (Figure 5B), betweenness (Figure 5C), stress (Figure 5D), degree (Figure 5E), EPC (Figure 5F), MNC (Figure 5G), and MCC (Figure 5H). Four genes (CCNB1, CDK1, CYCS, and STAT3) were consistently identified by all methods and selected as cHubGs, as illustrated in Figure 5I.

**Figure 4:**
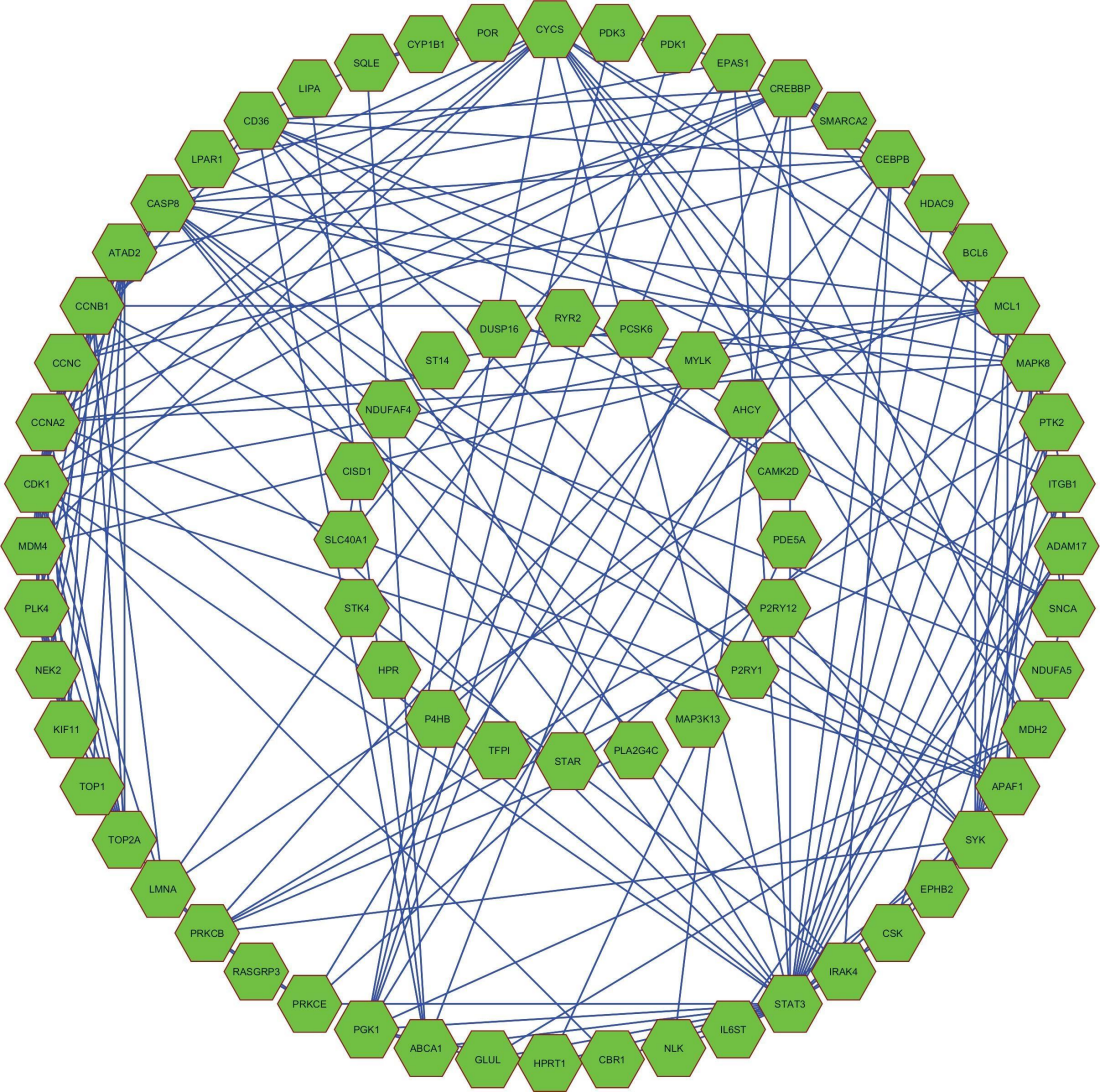
PPI network for identified cHDEGs.

**Figure 5:**
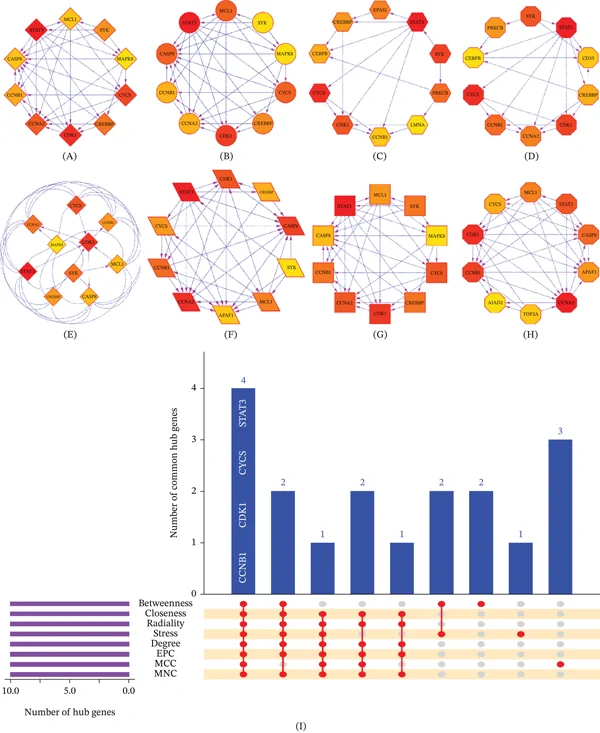
Network of identified HubGs of cHDEGs using eight CytoHubba plugins in (A) closeness, (B) radiality, (C) betweenness, (D) stress, (E) degree, (F) EPC, (G) MNC, (H) MCC, and (I) UpSet plot of cHubGs from eight algorithms.

### 3.3. Functional and Pathway Enrichment Analysis of cHDEGs

GO and KEGG pathway enrichment analyses were performed to characterize the biological functions and pathways associated with the cHDEGs. The top enriched GO terms in the BP, MF, and CC categories are presented in Figure 6A–C and Table S1.

**Figure 6:**
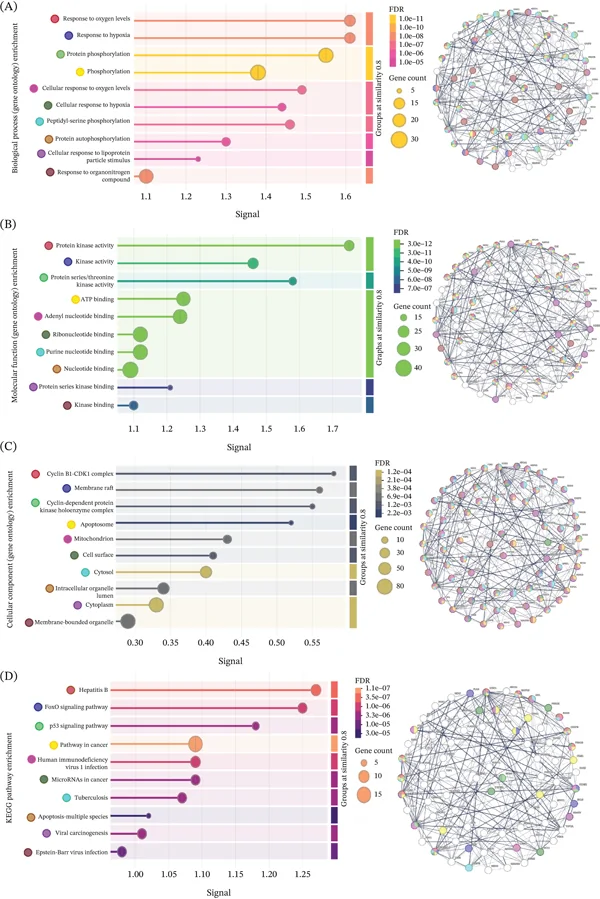
The top 10 significantly enriched GO terms (A) BP, (B) MF, (C) CC, and (D) KEGG pathways that are involved in the pathogenesis processes of NDs. Here, the point size and color depend on involved gene numbers and significance of the enriched analysis (FDR).

#### 3.3.1. GO Enrichment Analysis

##### 3.3.1.1. BP

The enriched BP terms included protein phosphorylation, response to oxygen levels, response to hypoxia, response to organonitrogen compound, peptidyl‐serine phosphorylation, protein autophosphorylation, cellular response to oxygen levels, cellular response to hypoxia, and cellular response to lipoprotein particle stimulus (Figure 6A; Table S1). Protein phosphorylation and phosphorylation‐related terms showed similar patterns, with approximately 30 genes associated with protein phosphorylation and a signal value of approximately 1.55, compared with approximately 1.38 for phosphorylation. Response to oxygen levels and response to hypoxia were also similar, each involving approximately 20 genes, with signal values of approximately 1.61. Cellular response to oxygen levels and peptidyl‐serine phosphorylation involved approximately 15 genes, with signal values of approximately 1.49 and 1.47, respectively. Cellular response to hypoxia and protein autophosphorylation each involved approximately 15 genes, with signal values of approximately 1.44 and 1.31, respectively. Response to organonitrogen compound involved approximately 30 genes and had a signal value of approximately 1.10. Cellular response to lipoprotein particle stimulus involved approximately five genes, with a signal value of approximately 1.23.

##### 3.3.1.2. MF

The top enriched MF terms included protein kinase activity, ATP binding, adenyl nucleotide binding, purine nucleotide binding, ribonucleotide binding, nucleotide binding, kinase activity, protein serine/threonine kinase activity, kinase binding, and protein serine kinase binding (Figure 6B; Table S1). Protein kinase activity involved approximately 30 genes and had a signal value of approximately 1.75. The nucleotide‐binding terms showed similar enrichment patterns. ATP binding, adenyl nucleotide binding, purine nucleotide binding, ribonucleotide binding, and nucleotide binding involved approximately 40 genes each, with signal values of approximately 1.25, 1.24, 1.12, 1.12, and 1.09, respectively. Kinase activity involved approximately 30 genes, with a signal value of approximately 1.46, whereas protein serine/threonine kinase activity involved approximately 25 genes, with a signal value of approximately 1.58. Kinase binding involved approximately 30 genes and had a signal value of approximately 1.10. Protein serine kinase binding involved approximately 15 genes, with a signal value of approximately 1.21.

##### 3.3.1.3. CC

The top enriched CC terms were cytoplasm, cytosol, mitochondrion, membrane‐bounded organelle, membrane raft, intracellular organelle lumen, cyclin‐dependent protein kinase holoenzyme complex, cell surface, cyclin B1–CDK1 complex, and apoptosome (Figure 6C; Table S1). Cytoplasm and cytosol showed similar patterns, each involving approximately 80 genes. The signal values were approximately 0.33 for cytoplasm and 0.40 for cytosol. Mitochondrion and membrane raft each involved approximately 30 genes, with signal values of approximately 0.43 and 0.56, respectively. Cyclin B1–CDK1 complex and cyclin‐dependent protein kinase holoenzyme complex each involved approximately 10 genes, with signal values of approximately 0.58 and 0.55, respectively. Membrane‐bounded organelle involved approximately 80 genes, with a signal value of approximately 0.23. Intracellular organelle lumen involved approximately 50 genes, with a signal value of approximately 0.34, whereas cell surface involved approximately 30 genes, with a signal value of approximately 0.41. Apoptosome involved approximately 10 genes and had a signal value of approximately 0.52.

#### 3.3.2. KEGG Pathway Enrichment Analysis

KEGG pathway enrichment analysis identified the top 10 enriched pathways associated with the cHDEGs (Figure 6D; Table S2). These included pathways in cancer, hepatitis B, FoxO signaling pathway, human immunodeficiency virus 1 infection, miRNAs in cancer, tuberculosis, p53 signaling pathway, viral carcinogenesis, Epstein–Barr virus infection, and apoptosis—multiple species. FoxO signaling and human immunodeficiency virus 1 infection showed similar patterns, each involving approximately 10 genes, with signal values of approximately 1.25 and 1.09, respectively. The miRNAs in cancer, tuberculosis, p53 signaling, and viral carcinogenesis pathways each involved approximately 10 genes, with signal values of approximately 1.09, 1.07, 1.18, and 1.01, respectively. Pathways in cancer involved approximately 15 genes and had a signal value of approximately 1.09. Hepatitis B and Epstein–Barr virus infection each involved approximately 10 genes, with signal values of approximately 1.27 and 0.98, respectively. Apoptosis—multiple species involved approximately five genes and had a signal value of approximately 1.02.

### 3.4. Interaction Analysis of cHDEGs With Their Regulatory Factors

The regulatory interaction network of cHDEGs with TFs and miRNAs is presented in Figure 7A,B and Tables S3 and S4. These regulatory factors are involved in gene regulation at transcriptional and posttranscriptional levels. The TF interaction network included SP1, STAT3, MYC, TP53, BRCA1, E2F1, YBX1, PTTG1, NR4A1, and E2F3 (Figure 7A). SP1 showed comparatively lower significance and was associated with nine cHDEGs. In contrast, E2F1 was identified as the most significant TF and was also associated with nine cHDEGs, including the hub genes CDK1 and CCNB1. STAT3, MYC, BRCA1, YBX1, PTTG1, and NR4A1 showed greater significance than SP1, whereas TP53 and E2F3 showed lower significance than E2F1. The miRNA–cHDEG interaction network is shown in Figure 7B. Hsa‐miR‐92a‐3p was identified as a comparatively less significant miRNA and was associated with the largest number of genes (17), including three hub genes: CCNB1, CDK1, and STAT3. Hsa‐miR‐21‐5p was identified as the most significant miRNA, associated with 12 genes, including the hub gene STAT3. Hsa‐miR‐3190‐5p was the second most significant miRNA and was associated with nine genes, including CYCS. Based on the number of associated genes and FDR values, hsa‐miR‐24‐3p, hsa‐miR‐215‐5p, hsa‐miR‐132‐3p, hsa‐miR‐548n, hsa‐miR‐205‐5p, hsa‐miR‐200a‐3p, and hsa‐miR‐153‐3p were reported as more significant than hsa‐miR‐92a‐3p.

**Figure 7:**
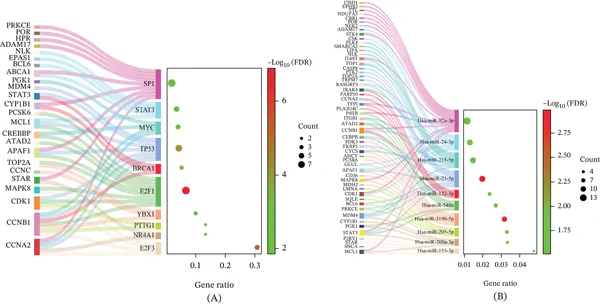
Scatter and Sankey plot of (A) TF and (B) miRNA. The color red represents high significance, and color green represents low significance.

### 3.5. Drug‐Likeness, ADMET, and Toxicity Analysis

A total of 164 turmeric‐derived compounds were collected and considered as potential ligands for the four target proteins, as illustrated in Figure 8. These compounds were evaluated using drug‐likeness criteria, Lipinski′s Ro5, ADMET predictions, and toxicity assessment. Following these screening steps, 77 compounds were retained for subsequent analyses (Table S5).

**Figure 8:**
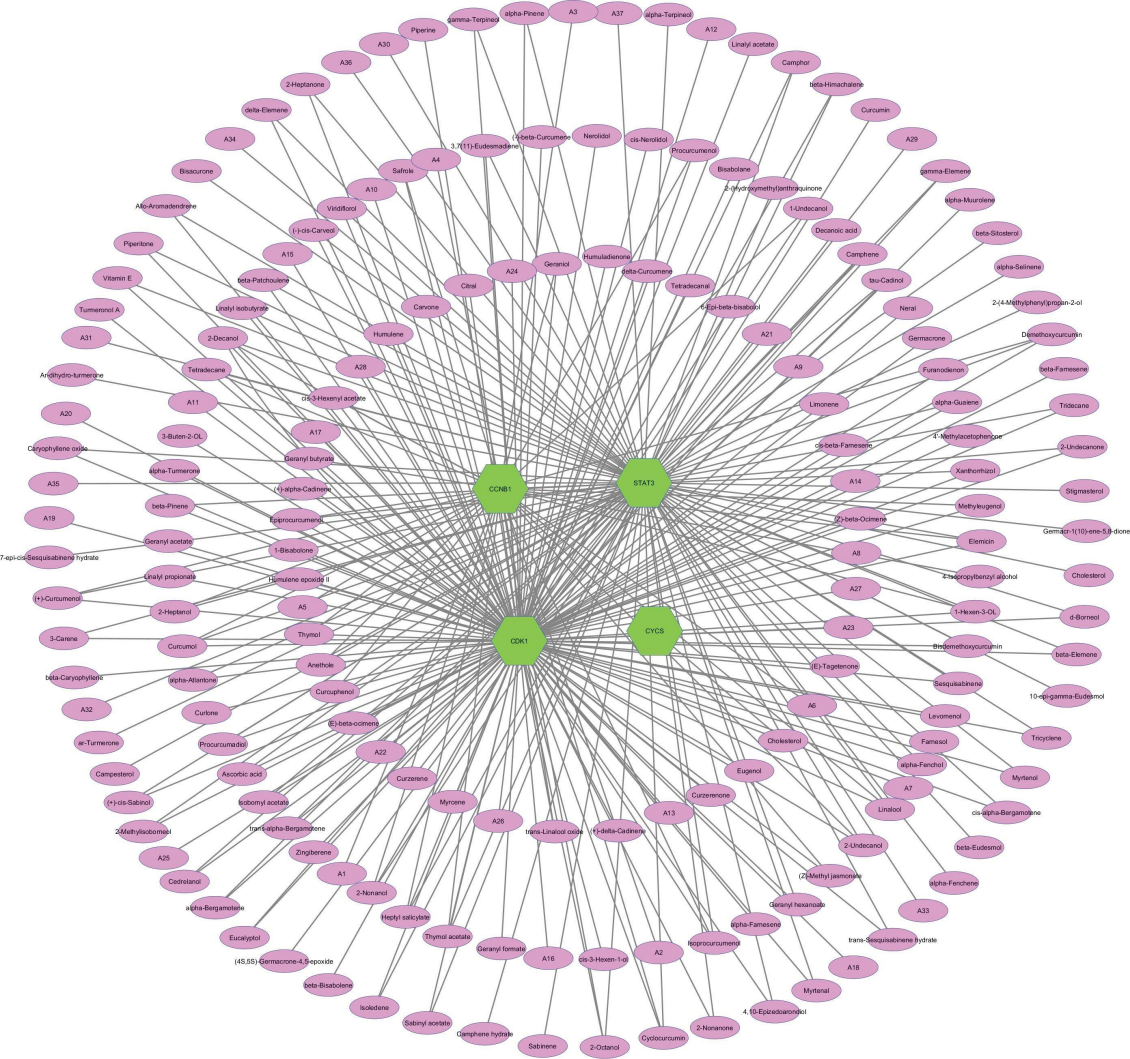
Network showing the relationships between cHDEGs and turmeric‐derived compounds.

### 3.6. Molecular Docking Analysis

The 3D structure of target proteins CDK1, STAT3, CCNB1, and CYCS were retrieved from PDB [72] with the PBD IDs 6GU2, 6QHD, 4YC3, and 5EXQ, respectively. The three‐dimensional structures of the 77 selected compounds were obtained from PubChem [67]. Molecular docking was performed for each compound against the four target proteins, and the results are presented in Figure 9A. The docking results for the compound–protein pairs with binding affinities meeting the specified threshold are presented in Figure 9B. Compounds with favorable binding affinity scores were prioritized, and the top‐ranked compound for each target protein was selected for further consideration. The four initially selected complexes were CDK1 + CID10469828, STAT3 + CID69879809, CCNB1 + CID5469424, and CYCS+CID69879809, with reported binding affinities of −8.4, −8.1, −8.9, and −9.1 kcal/mol, respectively. Table 2 presents the binding configurations of the four selected protein–ligand complexes, including the three‐dimensional conformations of the target proteins and the residues involved in the predicted interactions. Among the four complexes, CID69879809 was reported to show favorable binding with both STAT3 and CYCS. Because this compound was associated with two target proteins, the complex with the more favorable binding affinity was selected for further analysis. Accordingly, the CYCS + CID69879809 complex was retained. The three complexes selected for subsequent MDS were CDK1 + CID10469828, CCNB1 + CID5469424, and CYCS + CID69879809.

**Figure 9:**
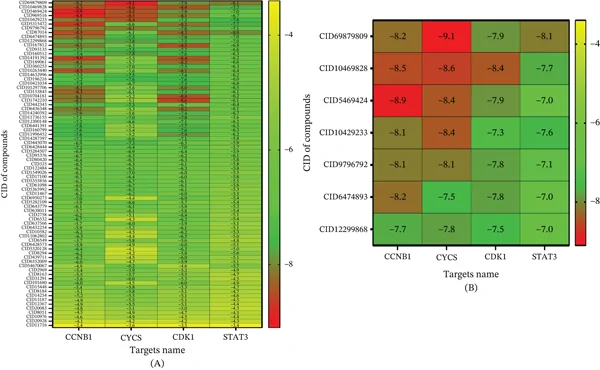
Binding affinity scores of the drug–protein complexes: (A) complete docking results and (B) complexes with affinities lower than or equal to −7.0 kcal/mol.

**Table 2:** Top‐ranked four drug‐target complexes highlighting their 3‐dimensional (3D) view and interacting residues.

Name of potential targets	Structure of compound	Binding affinity (kcal/mol)	3D structure of complex	2D diagram interaction	Interacting amino acids
CCNB1 (4YC3)	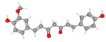 Demethoxycurcumin	−8.9	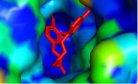	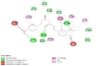	GLN132 LEU135 VA18 ILE10 PHE82 ALA31 VAL4 LEU55 PHE47 GLU51 LYS33 ALA145 LEU83 GLU81 ASN133
CDK1 (6GU2)	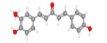 1E,4E)‐1‐(4‐Hydroxy‐3‐methoxyphenyl)‐5‐(4‐hydroxyphenyl)‐1,4‐pentadien‐3‐one	−8.4	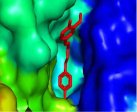	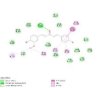	ILE10 LYS89 MET85 ASP86 LEU83 GLU81 ALA31 ALA145 PHE80 SER84 PHE82 LEU135 LYS33 VAL18 VAL64 ASP146 HE147 ASP146 LEU55 LEU78
STAT3 (6QHD)	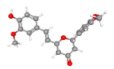 Cyclocurcumin	−8.1	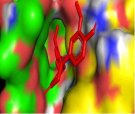	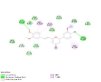	SER341 LEU378 GLY373S ER372 LEU436 HIS437 ASP369 VAL490 ALA377 LEU438 ASN491 ASN489 LYS495 ASN486 LYS488
CYCS (5EXQ)	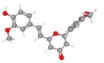 Cyclocurcumin	−9.1	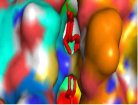	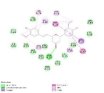	GLN132 LEU135 VAL18 ILE10 PHE82 ALA31 PHE80 ASP146 VAL64 LEU55 PHE147 GLU51 LYS33 ASN13

### 3.7. MDS Analysis

#### 3.7.1. RG Analysis

The Rg was used to assess changes in the compactness of the protein–ligand complexes during the 500‐ns molecular dynamics simulations. The Rg profiles of CCNB1 + CID5469424, CYCS + CID69879809, and CDK1 + CID10469828 are shown in Figure 10A. The CYCS + CID69879809 complex maintained Rg values below 1.4 nm throughout the simulation, indicating a relatively compact structure. The CDK1 + CID10469828 complex showed comparatively limited variation, with Rg values fluctuating between 2.02 and 2.08 nm. In contrast, the Rg of the CCNB1 + CID5469424 complex decreased from approximately 2.10 to 2.02 nm during the first 200 ns, then fluctuated between 2.02 and 2.09 nm before reaching approximately 2.10 nm at the end of the simulation.

**Figure 10:**
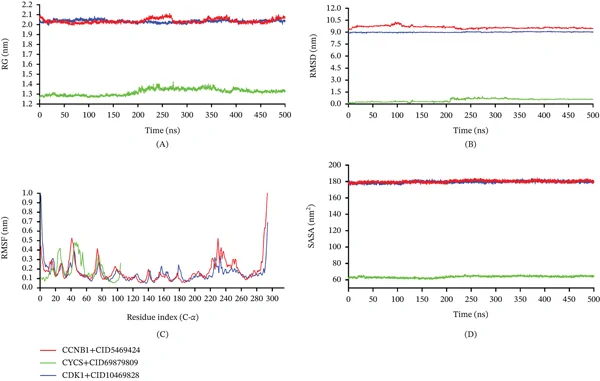
(A) RG, (B) RMSD, (C) RMSF, and (D) SASA analysis of the top‐ranked three drug–target complexes.

#### 3.7.2. RMSD Analysis

RMSD was used to examine structural deviations of the simulated complexes relative to a reference structure. The reported average RMSD values for CCNB1 + CID5469424, CYCS + CID69879809, and CDK1 + CID10469828 were 9.5, 0.75, and 9 nm, respectively. As shown in Figure 10B, the CCNB1 + CID5469424 complex displayed the poorest stability, with RMSD values increasing rapidly from 9.2 to 10 nm within the first 100 ns, then fluctuating between 9.8 and 9.3 nm before reaching 9.2 nm. The CDK1 + CID10469828 complex maintained average stability and had very little fluctuation. In comparison, the CYCS + CID69879809 is highly stable from 0 to 210 ns, then from 210 ns to the end of the simulation, there is a slight fluctuation that is in a suitable range (Figure 10B).

#### 3.7.3. RMSF Analysis

RMSF was used to characterize residue‐level flexibility within the simulated protein systems. Figure 10C shows the RMSF profiles across the C*α* residue indices, ranging from approximately 0 to 300, with RMSF values spanning approximately 0.1–1.0 nm. The CYCS + CID69879809 complex exhibited relatively low residue‐level fluctuations, with small peaks near Residues 20 and 90, reaching approximately 0.2 and 0.3 nm, respectively. The CDK1 + CID10469828 complex showed moderate fluctuations near Residues 40, 120, and 160, with peaks of approximately 0.45 nm, indicating localized flexibility. The CCNB1 + CID5469424 complex showed larger fluctuations, particularly across Residues 25–35, 220–240, and near Residue 290, with a maximum RMSF of approximately 0.75 nm. The CYCS + CID69879809 complex also showed a small peak around Residues 40–60, whereas the remaining residues exhibited relatively limited fluctuations.

#### 3.7.4. SASA Analysis

SASA was analyzed to characterize changes in solvent exposure of the protein–ligand complexes (Figure 10D). The SASA values for CCNB1 + CID5469424 and CDK1 + CID10469828 remain consistently around 180 nm^2^, reflecting stable solvent accessibility and structural robustness throughout the simulation. In contrast, CYCS + CID69879809 maintains a markedly lower SASA of approximately 60 nm^2^, indicating a more compact conformation with reduced solvent exposure, suggestive of strong protein–ligand interactions. Collectively, the stable SASA trends across all three complexes highlight their favorable binding behavior and overall structural stability.

#### 3.7.5. PCA Analysis of Trajectories

PCA was performed on three datasets to characterize their structural landscapes, revealing distinct patterns of group separation and variance distribution across multiple principal component projections, as indicated in Figure 11A–C. CCNB1 + CID5469424 exhibited clear clustering separation, with the first three principal components accounting for 52.39% of the variance (PC1: 28.83%, PC2: 15.86%, and PC3: 7.27%). All projections showed distinct clustering: PC1 versus PC2 displayed clear separation along both axes; PC1 versus PC3 maintained distinct clusters with minimal overlap; and PC2 versus PC3 showed well‐separated groups. This consistent clustering across all views indicates two well‐defined conformational states with discrete transitions rather than continuous flexible sampling. CDK1 + CID10469828 exhibited partial clustering separation, with the first three principal components accounting for 46.66% of the variance (PC1: 31.94%, PC2: 8.72%, and PC3: 6%). All projections showed moderate clustering: PC1 versus PC2 displayed diagonal separation with overlapping transition regions; PC1 versus PC3 maintained partial separation with significant overlap in central areas; and PC2 versus PC3 showed mixed distributions with intermediate overlap. This clustering pattern across all views indicates conformational states with some separation but substantial transitional sampling, suggesting moderate structural flexibility with semidiscrete conformational transitions. CYCS + CID69879809 exhibited excellent clustering separation, with the first three principal components accounting for 78% of the variance (PC1: 68.65%, PC2: 5.84%, and PC3: 3.51%). All projections showed distinct clustering: PC1 versus PC2 displayed clear horizontal separation along PC1 with minimal overlap; PC2 versus PC3 showed overlapping distributions with less distinct separation; and PC1 versus PC3 maintained clear separation primarily along the PC1 axis. This clustering pattern indicates two highly distinct conformational states with discrete transitions, with PC1 capturing the primary conformational difference between states.

**Figure 11:**
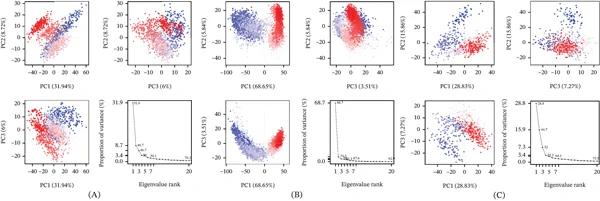
PCA analysis of (A) CCNB1 + CID5469424, (B) CDK1 + CID10469828, and (C) CYCS + CID69879809.

#### 3.7.6. DCCM Analysis of Trajectories

DCCM analysis was performed to characterize correlated residue motions in the three protein–ligand complexes (Figure 12A–C). CCNB1 + CID5469424 exhibits strong positive correlations along the diagonal, particularly at Residues 25–75, 125–175, and 225–275, indicating coordinated intradomain motions. Anticorrelated regions appear as off‐diagonal elements throughout the matrix, notably between the ranges 75–125 and 125–175, suggesting opposing interdomain movements. The block‐like pattern highlights well‐defined dynamic domains with cooperative and anticooperative motions, supporting complex conformational transitions. CDK1 + CID10469828 shows strong positive correlations along the diagonal at Residues 50–80, 100–130, 180–220, and 240–270, indicating coordinated intradomain motions. Anticorrelated regions appear as off‐diagonal elements, notably between Residues 50–100 and 150–200, and between Residues 100–150 and 200–250, suggesting opposing interdomain movements. The block‐like pattern reveals well‐defined dynamic domains with cooperative and anticooperative motions, supporting complex conformational transitions. CYCS + CID69879809 shows strong positive correlations along the diagonal at Residues 1–35 and 75–100, indicating coordinated intradomain motions. Anticorrelated regions appear as off‐diagonal elements, notably between Residues 1–35 and 75–100, and between Residues 35–50 and 50–75, suggesting opposing interdomain movements. The block‐like pattern reveals well‐defined dynamic domains with cooperative and anticooperative motions, supporting complex conformational transitions.

**Figure 12:**
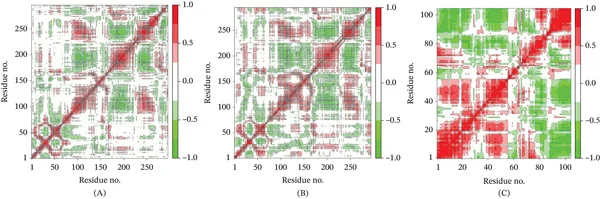
DCCM analysis of (A) CCNB1 + CID5469424, (B) CDK1 + CID10469828, and (C) CYCS + CID69879809.

#### 3.7.7. FEL Analysis Trajectories

The FEL of the three protein–ligand complexes, projected onto PC1 and PC2, revealed distinct thermodynamic profiles (Figure 13A–C). For CDK1–CID10469828 (Residues 0–290), polyproline segments were observed at Residues 40–60, 140–160, and 240–260. The pronounced flexibility indicates ligand‐stabilized conformational selection, consistent with the monomodal FEL and a single convergent binding mode. The CCNB1–CID5469424 complex (Residues 0–290) exhibited polyproline regions at 50–70, 150–170, and 240–260, with persistent disordered segments suggesting ligand‐induced disorder retention. This observation aligns with the bimodal FEL, representing two kinetically isolated binding modes with negligible interconversion. In the CYCS–CID69879809 complex (Residues 0–104), transient polyproline structures appeared at 20–30, 60–70, and 90–100, reflecting ligand‐driven conformational flexibility. This dynamic behavior corresponds to the bimodal FEL, indicative of two high‐affinity conformational states in equilibrium. Collectively, ligand binding was found to preserve intrinsic disorder across all complexes, facilitating conformational selection through distinct mechanism selection in CDK1, retention in CCNB1, and flexibility in CYCS. These structural dynamics directly correlate with the FEL topologies, reinforcing the presence of diverse, high‐affinity recognition pathways in each complex.

**Figure 13:**
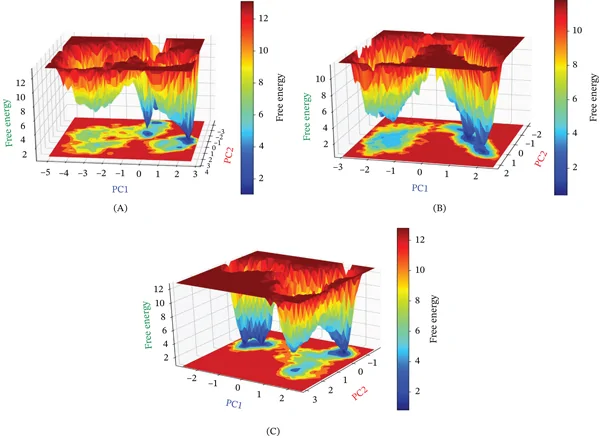
FEL analysis of (A) CCNB1 + CID5469424, (B) CDK1 + CID10469828, and (C) CYCS + CID69879809.

#### 3.7.8. SSA Analysis of Trajectories

The secondary structure profiles of the three protein–ligand complexes were examined throughout the 500‐ns simulations (Figure 14A–C). The reported profiles were predominantly characterized by bend (purple) and coil/loop (gray) regions, with sparse and transient polyproline helix (red) segments. Stable *α*‐helical and *β*‐sheet structures were not prominently observed in the reported profiles.

**Figure 14:**
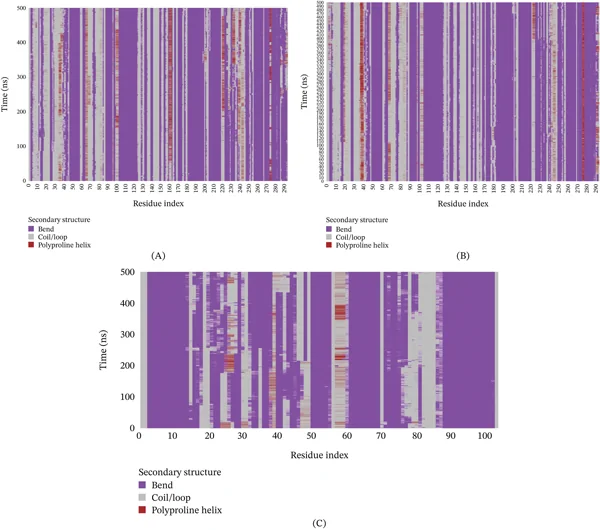
SSA analysis of (A) CCNB1 + CID5469424, (B) CDK1 + CID10469828, and (C) CYCS + CID69879809.

## 4. Discussion

Despite the promising molecular interactions identified in the present study, the translational applicability of turmeric‐derived compounds requires careful consideration. Turmeric has a long history of traditional medicinal use; however, several of its major bioactive constituents have encountered substantial challenges in their development as clinically useful therapeutic agents. In particular, limited bioavailability, absorption and solubility issues, rapid metabolism, and pharmacokinetic uncertainties may restrict the ability of these compounds to achieve and maintain therapeutically relevant concentrations in vivo. Therefore, favorable binding affinities, stable molecular dynamics trajectories, network‐based target associations, and predicted ADMET profiles should not be interpreted as direct evidence of therapeutic efficacy or clinical applicability. Computational approaches, including network analysis, molecular docking, molecular dynamics simulations, and in silico ADMET prediction, are valuable hypothesis‐generating tools, but their predictions require validation through appropriate biochemical, cellular, animal, pharmacokinetic, and ultimately clinical studies. Moreover, target validation, the predictive accuracy of preclinical models, disease heterogeneity, patient‐to‐patient variability, and clinical attrition represent important barriers to successful drug development. As emphasized by Dsouza [73], promising preclinical or computational findings do not necessarily translate into successful clinical therapies. Accordingly, the turmeric‐derived compounds identified in the present study should be regarded as potential candidates for further experimental investigation rather than as clinically validated therapeutic agents.

The identification of shared genes in this study was based on the overlap of disease‐specific DEG sets derived from four independent transcriptomic datasets. Although this approach provides a straightforward strategy for identifying genes consistently dysregulated across the studied neurological disorders, differences in tissue origin, experimental platforms, sample characteristics, and potential batch effects may influence the individual DEG profiles and, consequently, the shared gene set. The selected criteria were applied consistently across datasets to identify statistically significant and biologically relevant expression changes. Nevertheless, alternative thresholds or independent datasets may yield partially different shared gene profiles. Therefore, the identified cHDEGs should be considered candidate shared molecular signatures that warrant validation in independent cohorts and experimental systems.

NDs represent a major global health challenge, contributing substantially to mortality, disability, and healthcare burden worldwide. Among these disorders, AD, PD, GBM, and stroke are among the most prevalent and devastating conditions, characterized by complex pathological mechanisms and limited therapeutic options [74]. Although these disorders exhibit distinct clinical manifestations, increasing evidence suggests that they share common molecular abnormalities and biological pathways. In the present study, an integrative bioinformatics and network pharmacology approach was employed to identify the shared molecular signatures underlying AD, PD, GBM, and stroke and to explore turmeric‐derived bioactive compounds as potential multitarget therapeutic candidates.

Functional enrichment analysis revealed that the identified common differentially expressed genes (cHDEGs) were significantly associated with BP, CC, and MF involved in neurological disease progression. Notably, response to hypoxia, protein phosphorylation, peptidyl‐serine phosphorylation, cellular response to lipoprotein particle stimulus, and response to organonitrogen compounds were among the major enriched BPs. These findings suggest that the identified genes participate in several interconnected pathological mechanisms that contribute to the development and progression of multiple NDs.

Hypoxia was one of the major enriched BP identified in our analysis, suggesting its role as a shared pathogenic mechanism among AD, PD, GBM, and stroke. Hypoxic stress contributes to AD progression through oxidative stress, neuroinflammation, and neuronal dysfunction [75], whereas hypoxia‐induced alterations have also been linked to Parkinsonian symptoms [76]. In GBM, hypoxia regulates tumor aggressiveness, angiogenesis, invasion, and therapeutic resistance [77], whereas poststroke hypoxia is associated with neuronal injury and poor clinical outcomes [78]. Protein phosphorylation was another significantly enriched process, highlighting its importance in regulating neuronal function and disease progression. Abnormal phosphorylation events contribute to AD pathology [79], promote *α*‐synuclein aggregation in PD [80], regulate tumor growth and survival in GBM [81], and influence ischemic injury in stroke [82]. Similarly, serine phosphorylation and Pin1‐mediated signaling have been implicated in neurodegenerative mechanisms [83–85]. Additionally, lipoprotein‐related pathways were enriched, supporting the role of lipid dysregulation as a common feature of neurological disorders. Altered lipid transport and cholesterol metabolism are associated with AD risk [86], whereas lipoprotein‐mediated mechanisms have also been implicated in PD, GBM, and stroke pathogenesis [87–89]. Therefore, these findings suggest that hypoxia and cellular stress may interact with aberrant protein phosphorylation and lipid dysregulation to influence neuronal survival, cellular signaling, and tissue homeostasis, thereby contributing to convergent pathological mechanisms across AD, PD, GBM, and stroke.

CC enrichment analysis revealed significant involvement of the cyclin B1–CDK1 complex, cyclin‐dependent protein kinase holoenzyme complex, apoptosome, mitochondria, cytoplasm, and other intracellular organelles, indicating the importance of cell cycle regulation, apoptosis, and mitochondrial function in the shared pathogenesis of AD, PD, GBM, and stroke. Abnormal regulation of cyclin B1 and CDK1 signaling has been reported in neurological disorders and cancer. In AD and stroke, abnormal accumulation of cyclin B1 in affected brain regions contributes to neuronal dysfunction, whereas the CDK1/cyclin B complex is a critical regulator of mitosis and tumor progression in GBM [90]. Dysregulation of cyclin‐dependent kinases (CDKs) can disrupt cell cycle control and contribute to diverse pathological conditions, including neurodegeneration and cancer [91, 92]. The apoptosome was another important enriched CC, highlighting the central role of apoptosis in neurological disease progression. Apoptosome activation regulates neuronal cell death, with apoptotic pathways implicated as major contributors to neuronal loss in AD, PD, and stroke [93–95]. In GBM, altered apoptosome formation through APAF1 dysregulation may influence tumor cell survival and resistance to apoptosis [96]. Furthermore, mitochondrial dysfunction was strongly associated with the identified CC. Impaired mitochondrial activity, oxidative stress, calcium imbalance, and apoptosis are key pathological features of major neurodegenerative disorders, including AD, PD, and stroke [97, 98]. In GBM, mitochondrial DNA alterations contribute to abnormal energy metabolism, reactive oxygen species production, and resistance to chemotherapy‐induced apoptosis [99]. Collectively, these findings suggest that dysregulated cell‐cycle control, mitochondrial dysfunction, and apoptotic signaling are functionally interconnected, with disturbances in cellular energy and survival mechanisms potentially contributing to neuronal injury in AD, PD, and stroke and altered cell survival and proliferation in GBM.

MF enrichment analysis demonstrated significant involvement of protein kinase activity, serine/threonine kinase activity, ATP binding, nucleotide binding, and kinase‐associated functions, highlighting the importance of signal transduction, energy metabolism, and cellular regulation in the shared pathogenesis of AD, PD, GBM, and stroke. Protein kinases are key regulators of neuronal signaling and disease progression; dysregulated kinase activity contributes to inflammation, synaptic dysfunction, and tau hyperphosphorylation in AD [100]. Similarly, stress‐activated c‐Jun N‐terminal kinases (JNKs) are implicated in PD pathogenesis through responses to cellular stress, cytokines, and inflammatory signals [101]. Alterations in major kinase pathways, including PI3K/AKT/mTOR signaling, have also been associated with neurodegeneration and neurological disorders [102]. ATP‐ and nucleotide‐binding functions further indicate the involvement of metabolic dysregulation in these disorders. Mitochondria generate cellular energy through oxidative phosphorylation, and impaired ATP production is a key feature of neurodegenerative diseases [103]. In GBM, abnormal extracellular ATP accumulation contributes to tumor growth and microenvironmental regulation [104], while ATP‐binding cassette (ABC) transporters play important roles in ischemic brain injury [105]. Moreover, disturbances in nucleotide and purine metabolism have been linked to AD, PD, and GBM, suggesting their involvement in neuronal dysfunction and tumor progression [106, 107]. Dysregulation of protein kinases, including CaMKII and JNK3, further connects oxidative stress, mitochondrial dysfunction, neuroinflammation, and neuronal injury across multiple neurological disorders. Together, these findings suggest that altered kinase signaling and nucleotide metabolism may be functionally linked through cellular energy and signal‐transduction networks. Such dysregulation could influence oxidative stress responses, mitochondrial function, inflammation, and cell survival, providing a mechanistic link between the shared molecular abnormalities observed across AD, PD, GBM, and stroke.

KEGG pathway enrichment analysis revealed significant associations with several pathways involved in inflammation, apoptosis, cancer progression, immune regulation, and cellular stress responses, including the FoxO signaling pathway, p53 signaling pathway, pathways in cancer, miRNA‐related pathways, and viral infection‐associated pathways. These findings suggest that common regulatory networks contribute to the pathogenesis of AD, PD, GBM, and stroke. Viral infection‐related pathways, such as hepatitis B and human immunodeficiency virus (HIV) infection, have been linked to neurological complications, as viral‐mediated inflammation and immune dysregulation may contribute to neurodegenerative processes [108, 109]. The FoxO and p53 signaling pathways emerged as important regulators connecting neuronal survival, apoptosis, oxidative stress, and tumor progression. FOXO transcription factors (TFs) play essential roles in AD, PD, and stroke by regulating stress responses, metabolism, and neuronal homeostasis [110]. In GBM, FOXO1 represents a potential therapeutic target because of its involvement in growth factor receptor signaling and tumor cell survival [111]. Similarly, dysregulation of p53‐associated pathways contributes to neuronal damage and tumor development, making p53 signaling an important mechanism shared among AD, PD, GBM, and stroke [112, 113]. Furthermore, miRNA‐associated pathways were enriched, highlighting the importance of posttranscriptional regulation in neurological disorders and cancer. Specific miRNAs involved in endocytic regulation have been implicated in both neurodegeneration and tumor invasion, suggesting their potential as therapeutic targets [114]. Apoptosis‐related pathways were also identified, consistent with the established role of excessive programmed cell death in acute and chronic neurological disorders [115]. Overall, these findings indicate that dysregulated stress responses, kinase signaling, mitochondrial function, apoptosis, immune signaling, and posttranscriptional regulation form interconnected molecular networks rather than isolated pathways. In this framework, hypoxia and oxidative stress may influence kinase‐ and mitochondria‐dependent signaling, which can subsequently modulate apoptotic and cell‐survival pathways, whereas FoxO and p53 signaling may act as important regulatory nodes linking these processes. Such interconnected pathway perturbations may underlie the shared molecular signatures identified across AD, PD, GBM, and stroke despite their distinct pathological characteristics.

TF enrichment analysis identified several key regulatory molecules, including SP1, STAT3, MYC, TP53, BRCA1, E2F1, YBX1, PTTG1, NR4A1, and E2F3, suggesting their potential roles in regulating shared pathogenic pathways among AD, PD, GBM, and stroke. Among these, SP1 emerged as an important regulator of neurological disease‐associated genes. SP1 modulates the expression of proteins involved in AD pathology [116], regulates monoamine oxidase B (MAO‐B)–related pathways relevant to PD management [117], and exhibits context‐dependent effects following ischemic stroke [118]. Furthermore, increased SP1 expression in glioma cells suggests its contribution to tumor progression [119]. Other identified TFs also demonstrate cross‐disease regulatory functions. MYC has been implicated in neuronal repair mechanisms during neurodegenerative conditions [120], whereas BRCA1 may contribute to AD pathogenesis through regulation of oxidative DNA damage responses and is also associated with glioma progression and ischemic stroke [121, 122]. E2F family members, particularly E2F1, play critical roles in neuronal dysfunction, cell cycle regulation, and disease progression, with reported involvement in AD, PD, stroke, and GBM [123–125]. Additionally, NR4A1 exhibits disease‐specific regulatory functions, including altered expression in PD and AD, activation following cerebral ischemic injury, and oncogenic activity in GBM [126–128]. Therefore, these findings indicate that dysregulated transcriptional networks involving stress response, DNA repair, cell cycle control, inflammation, and tumor‐associated signaling may represent common regulatory mechanisms linking neurodegenerative diseases and brain tumors.

The miRNA regulatory network analysis identified several key miRNAs, including hsa‐miR‐92a‐3p, hsa‐miR‐24‐3p, hsa‐miR‐215‐5p, hsa‐miR‐21‐5p, hsa‐miR‐32‐3p, hsa‐miR‐548n, hsa‐miR‐3190‐5p, hsa‐miR‐205‐5p, hsa‐miR‐200a‐3p, and hsa‐miR‐153‐3p, suggesting their potential involvement in the shared molecular regulation of AD, PD, GBM, and stroke. miRNAs are critical posttranscriptional regulators that influence neuronal survival, inflammation, apoptosis, and tumor progression. Among the identified miRNAs, hsa‐miR‐92a‐3p has been associated with altered expression patterns in AD and PD and has also been validated as a potential biomarker in ischemic stroke [129–131]. Similarly, hsa‐miR‐24‐3p and hsa‐miR‐215‐5p have been implicated in neurodegenerative processes, with altered expression reported in AD and PD, while miR‐24‐3p also regulates proliferative responses in GBM cells [132–136]. Furthermore, hsa‐miR‐21‐5p showed relevance across neurological disorders, with altered expression observed in AD and stroke and regulatory effects on GBM cell behavior [137–139]. Other identified miRNAs, including hsa‐miR‐205‐5p, hsa‐miR‐200a‐3p, and hsa‐miR‐153‐3p, have also been associated with glioma progression or neurodegenerative mechanisms [140–144]. Overall, these findings highlight the importance of miRNA‐mediated posttranscriptional regulation as a common molecular layer connecting neurodegeneration and brain tumor biology. The identified miRNAs may serve as potential regulatory biomarkers or therapeutic targets for modulating shared pathogenic pathways among AD, PD, GBM, and stroke.

The use of multiple CytoHubba algorithms provided additional confidence in the prioritization of the cHubGs, as CCNB1, CDK1, CYCS, and STAT3 were consistently identified across the applied network‐based approaches. The cHubGs suggest their central roles in regulating shared pathogenic mechanisms among AD, PD, GBM, and stroke. The CCNB1 and CDK1, major regulators of cell cycle progression, have been reported to exhibit altered expression in AD, PD, and brain tumors, indicating their involvement in both neurodegenerative processes and tumor‐associated cellular dysregulation [145, 146]. CDK1 has also been identified as an important mediator of ischemia‐induced neuronal death, highlighting its contribution to stroke pathology [147]. Furthermore, CYCS, a key mitochondrial apoptotic regulator, has been implicated in AD, with altered expression observed following amyloid‐*β* treatment in neuronal models [148]. Increased CYCS expression has also been reported in MPTP‐induced PD models, suggesting its involvement in mitochondrial‐mediated neuronal injury [149]. STAT3, a major transcriptional regulator of inflammation, cell survival, and immune responses, represents another critical hub in the shared molecular network. Dysregulated STAT3 activation has been observed in AD, PD, and stroke models, linking inflammatory signaling with neuronal dysfunction [150]. In GBM, extensive evidence from cellular and animal models indicates that STAT3 contributes to tumor initiation, progression, and therapeutic resistance [151]. Therefore, these findings support the identified cHubGs as key molecular regulators connecting neurodegeneration, brain tumor progression, and ischemic injury, and they may represent promising targets for multidisease therapeutic strategies. They may also have potential utility as candidate biomarkers; however, their diagnostic value and therapeutic relevance require validation in independent patient cohorts and experimental models. Therefore, these computationally prioritized hub genes should not be considered experimentally validated biomarkers or therapeutic targets. Independent transcriptomic cohorts are needed to assess the reproducibility of these signatures, whereas appropriate in vitro and in vivo studies are required to confirm their biological functions and therapeutic potential.

Finally, molecular docking and molecular dynamics simulation analyses identified three promising turmeric‐derived bioactive compounds, namely, (1E,4E)‐1‐(4‐hydroxy‐3‐methoxyphenyl)‐5‐(4‐hydroxyphenyl)‐1,4‐pentadien‐3‐one, desmethoxycurcumin, and cyclocurcumin, which exhibited favorable binding interactions and stable complex formation with the identified therapeutic targets. Among these compounds, (1E,4E)‐1‐(4‐hydroxy‐3‐methoxyphenyl)‐5‐(4‐hydroxyphenyl)‐1,4‐pentadien‐3‐one and cyclocurcumin have previously been reported as naturally occurring constituents isolated from the rhizomes of *C. longa* [50], supporting their pharmacological relevance. Toxicity and ADMET analyses suggested that these compounds possess favorable safety profiles, with low predicted risks of neurotoxicity, hepatotoxicity, carcinogenicity, and cytotoxicity, while demonstrating potential BBB permeability. The ability of therapeutic compounds to cross the BBB is a critical consideration for neurological disease treatment, as it enables efficient delivery to the central nervous system and may improve therapeutic efficacy while minimizing peripheral adverse effects. Therefore, these findings support the potential of the identified turmeric‐derived compounds as candidates for further investigation in the context of neurological disorders, whereas acknowledging that their therapeutic efficacy and clinical relevance require experimental and clinical validation.

Overall, this study provides a computational framework for identifying shared molecular signatures and potential turmeric‐derived therapeutic candidates across AD, PD, GBM, and stroke. However, the identified hub genes and compounds require validation using independent transcriptomic cohorts and experimental approaches to confirm their biological functions, efficacy, and safety. In addition, MD stability assessments were primarily based on trajectory‐derived structural and dynamic metrics, and replicate simulations were not performed. Therefore, the observed differences among complexes should not be interpreted as definitive evidence of superior binding stability or therapeutic efficacy. Further in vitro, in vivo, and clinical investigations will therefore be essential to establish their therapeutic and translational potential.

## 5. Conclusions

This study integrated transcriptomic and network‐based analyses of Alzheimer′s disease, Parkinson′s disease, stroke, and GBM to identify shared molecular signatures and potential therapeutic targets. The identification of 81 cHDEGs and four cHubGs—CCNB1, CDK1, CYCS, and STAT3—highlighted molecular networks potentially involved in these disorders. Molecular docking and 500‐ns molecular dynamics simulations further identified desmethoxycurcumin, cyclocurcumin, and (1E,4E)‐1‐(4‐hydroxy‐3‐methoxyphenyl)‐5‐(4‐hydroxyphenyl)‐1,4‐pentadien‐3‐one as candidate compounds with favorable predicted binding interactions with the selected hub proteins. These findings provide a computational foundation for investigating shared disease mechanisms and turmeric‐derived compounds as potential multitarget therapeutic candidates. However, independent transcriptomic validation and further biochemical, in vitro, and in vivo studies are required to confirm the biological relevance of the cHubGs and assess the compounds′ safety and therapeutic efficacy before clinical application can be considered.

## Author Contributions

Conceptualization: Fee Faysal Ahmed. Data curation: Fee Faysal Ahmed, Sumi Rani Bayragi, and Riday Hossain. Formal analysis: Fee Faysal Ahmed, Riday Hossain, and Sumi Rani Bayragi. Investigation: Fee Faysal Ahmed, Riday Hossain, and Sumi Rani Bayragi. Methodology: Sumi Rani Bayragi, Riday Hossain, and Fee Faysal Ahmed. Project administration: Fee Faysal Ahmed. Software: Fee Faysal Ahmed, Riday Hossain, Md. Zohurul Islam, and Sumi Rani Bayragi. Supervision: Fee Faysal Ahmed. Validation: Fee Faysal Ahmed, Riday Hossain, and Sumi Rani Bayragi. Visualization: Riday Hossain and Fee Faysal Ahmed. Writing—original draft: Sumi Rani Bayragi, Riday Hossain, and Fee Faysal Ahmed. Writing—review and editing: Fee Faysal Ahmed, Riday Hossain, Md. Zohurul Islam, Sharmin Akter Sumi, and Sumi Rani Bayragi. Sumi Rani Bayragi, Riday Hossain, and Fee Faysal Ahmed have contributed equally to this work.

## Funding

This study was supported by Jashore University of Science and Technology (10.13039/501100016172; 25‐FoS‐06).

## Ethics Statement

The authors have nothing to report.

## Conflicts of Interest

The authors declare no conflicts of interest.

## Supporting Information

Additional supporting information can be found online in the Supporting Information section.

## Supporting information


**Supporting Information 1** Table S1: Top 10 highly enriched GO terms associated with cHDEGs.

**Supporting Information 2** Table S2: Top 10 highly enriched KEGG pathways associated with cHDEGs.

**Supporting Information 3** Table S3: Top 10 highly significant TFs for cHDEGs.

**Supporting Information 4** Table S4: Top 10 highly significant miRNAs for cHDEGs.

**Supporting Information 5** Table S5: Drug likeness property and toxicity analysis information.

## Data Availability

The data that support the findings of this study are available in National Center for Biotechnology Information at https://www.ncbi.nlm.nih.gov/. These data were derived from the following resources available in the public domain:- GSE22491, https://www.ncbi.nlm.nih.gov/geo/query/acc.cgi?acc=GSE22491. - GSE5281, https://www.ncbi.nlm.nih.gov/geo/query/acc.cgi?acc=GSE5281. - GSE122709, https://www.ncbi.nlm.nih.gov/geo/query/acc.cgi?acc=GSE122709. - GSE4290, https://www.ncbi.nlm.nih.gov/geo/query/acc.cgi?acc=GSE4290.
