# Supplementary material for: Integrative Transcriptomic and Network Analyses Reveal Common Molecular Signatures and Turmeric‐Derived Common Therapeutic Candidates for Neurological Diseases
Source: Biomed Res Int. 2026 Nov 4;2026:7855459. doi: 10.1155/bmri/7855459 (PMC13732813; doi:10.1155/bmri/7855459)
Supplement: Supplementary file 1 [file BMRI-2026-7855459-s002.docx]

**Table S4:** Top ten highly significant miRNAs for cHDEGs

| microRNAs | FDR | No. of cHDEGs | Name of cHDEGs |
| --- | --- | --- | --- |
| Hsa-miR-21-5p | 0.001085 | 12 | APAF1, RASGRP3, TRPM7, MDM4, FKBP5, PRKCE, TOP2A, PTK2, **STAT3,** BCL6, CEBPB, CASP8 |
| Hsa-miR-205-5p | 0.013101 | 6 | PRKCE, BCL6, SQLE, **CDK1,** LMNA, MDH2 |
| Hsa-miR-548n | 0.013101 | 7 | CEBPB, **CCNB1,** PGK1, ATAD2, **STAT3,** ITGB1, P4HB |
| Hsa-miR-92a-3p | 0.013101 | 16 | NEK2, STK4, TFPI, **CDK1,** POR, P4HB, **STAT3, CCNB1,** CBR1, NDUFA5, TTL, EPHB2, CISD1, IL6ST, MAPK8, MCL1 |
| Hsa-miR-215-5p | 0.016056 | 11 | ATAD2, ITGB1, TOP1, IL6ST, NLK, LIPA, MDM4, SMARCA2, IRAK4, PLK4, **CYCS** |
| Hsa-miR-153-3p | 0.030955 | 4 | MCL1, SNCA, STAR, P2RY1 |
| Hsa-miR-200a-3p | 0.030955 | 5 | **STAT3,** PGK1, MCL1, CYP1B1, MDM4 |
| Hsa-miR-132-3p | 0.030955 | 6 | PLA2G4C, TFPI, CCNA2, **CCNB1,** PARP10, IRAK4 |
| Hsa-miR-24-3p | 0.030955 | 11 | CCNA2, TOP1, **CDK1,** GLUL, **CCNB1,** CSK, MDM4, RASGRP3, APAF1, STK4, ADAM17 |

Note: **Bold** gene name of cHDEGs indicates the hub genes
