# Supplementary material for: Integrative Transcriptomic and Network Analyses Reveal Common Molecular Signatures and Turmeric‐Derived Common Therapeutic Candidates for Neurological Diseases
Source: Biomed Res Int. 2026 Nov 4;2026:7855459. doi: 10.1155/bmri/7855459 (PMC13732813; doi:10.1155/bmri/7855459)
Supplement: Supplementary file 2 [file BMRI-2026-7855459-s003.docx]

**Table S3:** Top ten highly significant TFs for cHDEGs

| TFs | FDR | No. of cHDEGs | Name of cHDEGs |
| --- | --- | --- | --- |
| E2F1 | 1.30E-07 | 9 | APAF1, ATAD2, **CCNB1**, **CDK1**, CREBBP, MAPK8, MCL1, PCSK6, TOP2A |
| E2F3 | 5.85E-06 | 4 | CCNA2, **CCNB1,** **CDK1,** MAPK8 |
| TP53 | 7.01E-05 | 7 | APAF1, CCNA2, **CCNB1,** **CDK1,** MCL1, MDM4, **STAT3** |
| SP1 | 0.001201 | 9 | ABCA1, ADAM17, APAF1, CCNA2, CYP1B1, HPR, POR, PRKCE, STAR |
| YBX1 | 0.003104 | 3 | **CCNB1,** CCNC, TOP2A |
| STAT3 | 0.003104 | 5 | ABCA1, BCL6, EPAS1, MCL1, NLK |
| MYC | 0.006718 | 4 | CCNA2, **CCNB1,** CREBBP, PGK1 |
| NR4A1 | 0.014506 | 2 | CCNA2, STAR |
| PTTG1 | 0.014506 | 2 | **CCNB1, CDK1** |
| BRCA1 | 0.014506 | 3 | **CCNB1,** CYP1B1, **STAT3** |

Note: **Bold** gene name of cHDEGs indicates the hub genes
