# Supplementary material for: Integrative Transcriptomic and Network Analyses Reveal Common Molecular Signatures and Turmeric‐Derived Common Therapeutic Candidates for Neurological Diseases
Source: Biomed Res Int. 2026 Nov 4;2026:7855459. doi: 10.1155/bmri/7855459 (PMC13732813; doi:10.1155/bmri/7855459)
Supplement: Supplementary file 3 [file BMRI-2026-7855459-s004.docx]

**Table S2:** Top ten highly enriched KEGG pathways associated with cHDEGs

| **KEGG term** | **FDR** | **No. of cHDEGs** | **Name of cHDEGs** |
| --- | --- | --- | --- |
| Pathways in cancer | 1.16E-06 | 15 | CREBBP, EPAS1, **STAT3**, **CYCS**, PTK2, CASP8, STK4, LPAR1, IL6ST, MAPK8, ITGB1, RASGRP3, APAF1, CCNA2, PRKCB |
| Hepatitis B | 4.57E-06 | 9 | CREBBP, **STAT3**, **CYCS**, CASP8, MAPK8, APAF1, IRAK4, CCNA2, PRKCB |
| FoxO signaling pathway | 8.55E-06 | 8 | **CCNB1**, CREBBP, **STAT3**, PLK4, STK4, MAPK8, BCL6, NLK |
| Human immunodeficiency virus 1 infection | 1.77E-05 | 9 | **CCNB1**, **CYCS,** LIMK2, PTK2, CASP8, **CDK1**, MAPK8, IRAK4, PRKCB |
| MicroRNAs in cancer | 2.79E-05 | 8 | CREBBP, **STAT3**, ST14, PRKCE, MDM4, MCL1, CYP1B1, PRKCB |
| Tuberculosis | 3.05E-05 | 8 | CREBBP, CEBPB, CYCS, CASP8, SYK, MAPK8, APAF1, IRAK4 |
| p53 signaling pathway | 3.81E-05 | 6 | **CCNB1, CYCS**, CASP8, MDM4, **CDK1**, APAF1 |
| Viral carcinogenesis | 4.83E-05 | 8 | CREBBP, **STAT3**, CASP8, SYK, IL6ST, **CDK1**, HDAC9, CCNA2 |
| Epstein-Barr virus infection | 6.06E-05 | 8 | **STAT3**, **CYCS**, CASP8, SYK, MAPK8, APAF1, IRAK4, CCNA2 |
| Apoptosis - multiple species | 0.00031 | 4 | **CYCS**, CASP8, MAPK8, APAF1 |

Note: **Bold** gene name of cHDEGs indicates the hub genes
