# Supplementary material for: Integrative Transcriptomic and Network Analyses Reveal Common Molecular Signatures and Turmeric‐Derived Common Therapeutic Candidates for Neurological Diseases
Source: Biomed Res Int. 2026 Nov 4;2026:7855459. doi: 10.1155/bmri/7855459 (PMC13732813; doi:10.1155/bmri/7855459)
Supplement: Supplementary file 4 [file BMRI-2026-7855459-s005.docx]

**Table S1:** Top ten highly enriched GO terms associated with cHDEGs

| GO terms | FDR | No. of cHDEGs | Name of cHDEGs |
| --- | --- | --- | --- |
| BP |  |  |  |
| Protein phosphorylation | 3.42E-11 | 24 | CSK, **CCNB1**, MAP3K13, PLK4, FER, PRKCE, LIMK2, PTK2, MYLK, TOP1, NEK2,  STK4, SYK, PDK1, CDK**1**, MAPK8, EPHB2, NLK, CAMK2D, IRAK4, CCNA2, TRPM7, PRKCB, PDK3 |
| phosphorylation | 5.36E-11 | 26 | CSK, **CCNB1**, **STAT3**, MAP3K13, PLK4, FER, PRKCE, LIMK2, PTK2, MYLK, TOP1, NEK2, STK4, PGK1, SYK, PDK1, **CDK1**, MAPK8, EPHB2, NLK, CAMK2D, IRAK4, CCNA2, TRPM7, PRKCB, PDK3 |
| Response to oxygen levels | 5.64E-09 | 15 | AHCY, CREBBP, EPAS1, PRKCE, ADAM17, P4HB, RYR2, MDM4, LMNA, PGK1, LPAR1, PDK1, APAF1, CCNA2, PDK3 |
| Response to hypoxia | 1.19E-08 | 14 | AHCY, CREBBP, EPAS1, PRKCE, ADAM17, P4HB, RYR2, MDM4, LMNA, PGK1, PDK1, APAF1, CCNA2, PDK3 |
| Response to organonitrogen compound | 2.65E-08 | 22 | CSK, GNAO1, **STAT3**, FER, HPRT1, P2RY1, CEBPB, PRKCE, P2RY12, PTK2, RYR2, ABCA1, SYK, **CDK1**, ITGB1, EPHB2, HDAC9, CD36, POR, CYP1B1, CCNA2, SNCA |
| peptidyl-serine phosphorylation | 3.19E-07 | 11 | MAP3K13, PRKCE, TOP1, STK4, SYK, CDK1, MAPK8, NLK, CAMK2D, PRKCB, PDK3 |
| Cellular response to oxygen levels | 4.71E-07 | 10 | EPAS1, PRKCE, P4HB, MDM4, LMNA, PGK1, LPAR1, PDK1, CCNA2, PDK3 |
| Cellular response to hypoxia | 1.30E-06 | 9 | EPAS1, PRKCE, P4HB, MDM4, LMNA, PGK1, PDK1, CCNA2, PDK3 |
| Protein autophosphorylation | 2.10E-06 | 10 | CSK, MAP3K13, FER, PTK2, NEK2, STK4, SYK, NLK, CAMK2D, TRPM7 |
| Cellular response to lipoprotein particle stimulus | 5.61E-05 | 5 | ADAM17, ABCA1, SYK, ITGB1, CD36 |
|  |  |  |  |
| CC |  |  |  |
| Cytoplasm | 0.0012 | 70 | AHCY, CSK, TFPI, **CCNB1**, KIF11, SLC40A1, CREBBP, EPAS1, **STAT3**, MAP3K13, SQLE, PLK4, STAR, FER, CBR1, HPRT1, DUSP16, P2RY1, CEBPB, PRKCE, CYCS, GLUL, ADAM17, MDH2, P4HB, LIPA, LIMK2, PTK2, SDC3, PDE5A, CASP8, MYLK, TOP1, RYR2, NEK2, LMNA, MCL1, NDUFAF4, STK4, PGK1, CISD1, LPAR1, ABCA1, SYK, PDK1, **CDK1**, MAPK8, ITGB1, EPHB2, BCL6, NLK, RASGRP3, HDAC9, TOP2A, CD36, NDUFA5, POR, CAMK2D, PARP10, FKBP5, BPNT1, APAF1, PLA2G4C, CYP1B1, IRAK4, CCNA2, PCSK6, PRKCB, PDK3, SNCA |
| Cytosol | 0.0013 | 43 | AHCY, CSK, CCNB1, KIF11, SLC40A1, EPAS1, **STAT3**, PLK4, FER, CBR1, HPRT1, DUSP16, PRKCE, CYCS, GLUL, ADAM17, P4HB, LIPA, PTK2, PDE5A, CASP8, MYLK, NEK2, LMNA, MCL1, STK4, PGK1, SYK, **CDK1**, MAPK8, EPHB2, NLK, POR, CAMK2D, PARP10, FKBP5, BPNT1, APAF1, PLA2G4C, IRAK4, CCNA2, PRKCB, SNCA |
| Membrane raft | 0.0054 | 9 | CSK, TFPI, ADAM17, CASP8, PGK1, ABCA1, IL6ST, ITGB1, CD36 |
| Mitochondrion | 0.0054 | 20 | **CCNB1,** STAR, P2RY1, PRKCE, **CYCS**, GLUL, MDH2, CASP8, MCL1, NDUFAF4,  CISD1, PDK1, **CDK1**, MAPK8, NDUFA5, POR, PLA2G4C, CYP1B1, PDK3, SNCA |
| Intracellular organelle lumen | 0.0054 | 42 | **CCNB1**, SLC40A1, CREBBP, EPAS1, STAT3, PLK4, STAR, ATAD2, DUSP16, CEBPB, **CYCS**, ADAM17, MDH2, P4HB, LIPA, SDC3, CASP8, TOP1, NEK2, MDM4, LMNA, MCL1, STK4, SMARCA2, PDK1, **CDK1**, MAPK8, EPHB2, BCL6, NLK, HDAC9, TOP2A, CAMK2D, CCNC, PARP10, FKBP5, APAF1, PLA2G4C, CCNA2, PCSK6, PRKCB, PDK3 |
| Membrane-bounded organelle | 0.0054 | 71 | AHCY, CSK, TFPI, CCNB1, KIF11, SLC40A1, CREBBP, EPAS1, **STAT3**, SQLE, PLK4, STAR, FER, ATAD2, CBR1, HPRT1, DUSP16, P2RY1, CEBPB, PRKCE, **CYCS**, GLUL, ADAM17, MDH2, P4HB, LIPA, LIMK2, PTK2, SDC3, CASP8, TOP1, RYR2, NEK2, MDM4, LMNA, MCL1, NDUFAF4, STK4, PGK1, CISD1, LPAR1, ABCA1, SYK, IL6ST, SMARCA2, PDK1, **CDK1**, MAPK8, ITGB1, EPHB2, BCL6, NLK, HDAC9, TOP2A, CD36, NDUFA5, POR, CAMK2D, CCNC, PARP10, HPR, FKBP5, APAF1, PLA2G4C, CYP1B1, IRAK4, CCNA2, PCSK6, PRKCB, PDK3, SNCA |
| Cyclin-dependent protein kinase holoenzyme complex | 0.0131 | 4 | **CCNB1, CDK1**, CCNC, CCNA2 |
| Cell surface | 0.0131 | 13 | TFPI, P2RY1, P2RY12, ADAM17, P4HB, SDC3, LPAR1, ABCA1, IL6ST, ITGB1,  EPHB2, CD36, PCSK6 |
| Cyclin B1-CDK1 complex | 0.0145 | 2 | **CCNB1, CDK1** |
| Apoptosome | 0.0224 | 2 | **CYCS**, APAF1 |
|  |  |  |  |
| MF |  |  |  |
| Protein kinase activity | 3.32E-12 | 22 | CSK, MAP3K13, PLK4, FER, PRKCE, LIMK2, PTK2, MYLK, TOP1, NEK2, STK4, SYK, PDK1, **CDK1**, MAPK8, EPHB2, NLK, CAMK2D, IRAK4, TRPM7, PRKCB, PDK3 |
| ATP binding | 3.32E-12 | 32 | CSK, TTL, KIF11, MAP3K13, PLK4, FER, ATAD2, P2RY1, PRKCE, GLUL, LIMK2, PTK2, MYLK, TOP1, NEK2, STK4, PGK1, ABCA1, SYK, SMARCA2, PDK1, **CDK1**, MAPK8, EPHB2, NLK, TOP2A, CAMK2D, APAF1, IRAK4, TRPM7, PRKCB, PDK3 |
| Adenyl nucleotide binding | 3.32E-12 | 33 | AHCY, CSK, TTL, KIF11, MAP3K13, PLK4, FER, ATAD2, P2RY1, PRKCE, GLUL, LIMK2, PTK2, MYLK, TOP1, NEK2, STK4, PGK1, ABCA1, SYK, SMARCA2, PDK1, **CDK1**, MAPK8, EPHB2, NLK, TOP2A, CAMK2D, APAF1, IRAK4, TRPM7, PRKCB, PDK3 |
| Nucleotide binding | 3.32E-12 | 38 | AHCY, CSK, TTL, KIF11, GNAO1, MAP3K13, SQLE, PLK4, FER, ATAD2, HPRT1, P2RY1, PRKCE, GLUL, LIMK2, PTK2, PDE5A, MYLK, TOP1, NEK2, STK4, PGK1, ABCA1, SYK, SMARCA2, PDK1, **CDK1**, MAPK8, EPHB2, NLK, TOP2A, POR, CAMK2D, APAF1, IRAK4, TRPM7, PRKCB, PDK3 |
| Purine nucleotide binding | 3.34E-12 | 35 | AHCY, CSK, TTL, KIF11, GNAO1, MAP3K13, PLK4, FER, ATAD2, P2RY1, PRKCE, GLUL, LIMK2, PTK2, PDE5A, MYLK, TOP1, NEK2, STK4, PGK1, ABCA1, SYK, SMARCA2, PDK1, **CDK1**, MAPK8, EPHB2, NLK, TOP2A, CAMK2D, APAF1, IRAK4, TRPM7, PRKCB, PDK3 |
| Ribonucleotide binding | 3.34E-12 | 35 | CSK, TTL, KIF11, GNAO1, MAP3K13, PLK4, FER, ATAD2, P2RY1, PRKCE, GLUL, LIMK2, PTK2, PDE5A, MYLK, TOP1, NEK2, STK4, PGK1, ABCA1, SYK, SMARCA2, PDK1, **CDK1**, MAPK8, EPHB2, NLK, TOP2A, POR, CAMK2D, APAF1, IRAK4, TRPM7, PRKCB, PDK3 |
| Kinase activity | 3.01E-11 | 23 | CSK, MAP3K13, PLK4, FER, PRKCE, LIMK2, PTK2, MYLK, TOP1, NEK2, STK4, PGK1, SYK, PDK1, **CDK1**, MAPK8, EPHB2, NLK, CAMK2D, IRAK4, TRPM7, PRKCB, PDK3 |
| Protein serine/threonine kinase activity | 7.48E-10 | 17 | MAP3K13, PLK4, PRKCE, LIMK2, MYLK, TOP1, NEK2, STK4, SYK, **CDK1**, MAPK8, NLK, CAMK2D, IRAK4, TRPM7, PRKCB, PDK3 |
| Kinase binding | 9.53E-08 | 19 | CSK, **CCNB1**, KIF11, STAT3, MAP3K13, FER, DUSP16, CEBPB, PTK2, RYR2, SYK, MAPK8, ITGB1, RASGRP3, HDAC9, TOP2A, IRAK4, CCNA2, PRKCB |
| Protein serine kinase activity | 7.75E-07 | 13 | MAP3K13, PLK4, PRKCE, LIMK2, NEK2, STK4, **CDK1**, MAPK8, NLK, CAMK2D, IRAK4, TRPM7, PRKCB |

Note: **Bold** gene name of cHDEGs indicates the hub genes
